# Impact of the Noise Penalty Factor on Quantification in Bayesian Penalized Likelihood (Q.Clear) Reconstructions of ^68^Ga-PSMA PET/CT Scans

**DOI:** 10.3390/diagnostics11050847

**Published:** 2021-05-08

**Authors:** Sjoerd Rijnsdorp, Mark J. Roef, Albert J. Arends

**Affiliations:** 1 Department of Medical Physics, Catharina Hospital Eindhoven, Michelangelolaan 2, 5623 EJ Eindhoven, The Netherlands; bertjan.arends@catharinaziekenhuis.nl; 2 Department of Nuclear Medicine, Catharina Hospital Eindhoven, Michelangelolaan 2, 5623 EJ Eindhoven, The Netherlands; mark.roef@catharinaziekenhuis.nl

**Keywords:** ^68^Ga-PSMA PET/CT, recovery coefficient, quantitative PET, Bayesian penalized likelihood

## Abstract

Functional imaging with ^68^Ga prostate-specific membrane antigen (PSMA) and positron emission tomography (PET) can fulfill an important role in treatment selection and adjustment in prostate cancer. This article focusses on quantitative assessment of ^68^Ga-PSMA-PET. The effect of various parameters on standardized uptake values (SUVs) is explored, and an optimal Bayesian penalized likelihood (BPL) reconstruction is suggested. PET acquisitions of two phantoms consisting of a background compartment and spheres with diameter 4 mm to 37 mm, both filled with solutions of ^68^Ga in water, were performed with a GE Discovery 710 PET/CT scanner. Recovery coefficients (RCs) in multiple reconstructions with varying noise penalty factors and acquisition times were determined and analyzed. Apparent recovery coefficients of spheres with a diameter smaller than 17 mm were significantly lower than those of spheres with a diameter of 17 mm and bigger (*p* < 0.001) for a tumor-to-background (T/B) ratio of 10:1 and a scan time of 10 min per bed position. With a T/B ratio of 10:1, the four largest spheres exhibit significantly higher RCs than those with a T/B ratio of 20:1 (*p* < 0.0001). For spheres with a diameter of 8 mm and less, alignment with the voxel grid potentially affects the RC. Evaluation of PET/CT scans using (semi-)quantitative measures such as SUVs should be performed with great caution, as SUVs are influenced by scanning and reconstruction parameters. Based on the evaluation of multiple reconstructions with different β of phantom scans, an intermediate β (600) is suggested as the optimal value for the reconstruction of clinical ^68^Ga-PSMA PET/CT scans, considering that both detectability and reproducibility are relevant.

## 1. Introduction

Prostate cancer is the most frequent occurring malignancy in men. Global incidence in 2015 was estimated at over 1.6 million with prostate cancer having the highest incidence of all cancers in Western Europe, United States and Canada [1]. Many prostate cancers have a relatively indolent behavior and do not lead to significant medical complaints during the lifetime of a patient. However, patients may eventually progress to metastatic and/or castration-resistant prostate cancer (CRPC), which is considered an incurable and fatal stage of the disease. The optimal treatment for metastatic prostate cancer depends on characteristics of the tumor and of the patient, and may consist of multiple modalities including hormone therapy, chemotherapy, radiation therapy, and radionuclide therapy [2]. Selection and adjustment of a treatment is strongly dependent on treatment response. Therefore, there is a need for a tool that provides quantitative, lesion-specific and observer-independent response evaluation. Functional metabolic imaging with radiolabeled ^68^Ga prostate-specific membrane antigen (PSMA) and positron emission tomography (PET) is potentially such a tool. Although there is a vast amount of literature on PSMA-PET in staging and restaging of prostate cancer, response evaluation using PSMA-PET is less well explored and a standardized quantitative approach still needs to be developed.

It is known that uptake measurements of radiolabeled tracers with in vivo PET are affected by many parameters, as demonstrated by experience with ^18^F-fluor deoxyglucose (FDG), and standardization prior to application as response parameter is required [3]. For ^18^F-FDG-PET, repeatabilities of around 10% on average and higher are reported [4,5,6,7]. Notwithstanding the differences in pharmacodynamics and pharmacokinetics between FDG and PSMA, this probably applies equally to PSMA-PET. Before quantification of PSMA uptake can be used as a biomarker or surrogate endpoint to identify response to treatment, and before we can design sufficiently powered response evaluation studies, a thorough understanding of parameters affecting the quantitative results is required. Uptake of FDG and PSMA differ due to pharmacodynamical differences [8,9,10]. Therefore, comparison of uptake measurements from scans with different ligands should be approached with caution.

The spatial resolution of PET imaging is limited due to inherent physical characteristics such as positron range and noncollinearity of annihilation photons. Combined with detector characteristics and image sampling effects caused by discretization of the continuous activity distribution by recording it in finite sized voxels, these result in spillover from structures with a high activity concentration to those with a low activity concentration and vice versa, referred to as partial volume effect (PVE) [11,12]. The PVE is particularly of interest when the object is smaller than 2–3 times the spatial resolution expressed by its full width at half maximum (FWHM) [13,14] which is typically around 4–5 mm for state-of-the-art PET/CT systems [15]. As prostate cancer recurrence often involves relatively small metastatic nodal lesions, these effects are of particular importance with respect to PSMA signal evaluation. Resolution recovery techniques such as point spread function (PSF) modelling can be applied in order to partly recover the true shape and uptake of these lesions. In this study, attention is given in particular to image reconstruction using a Bayesian penalized likelihood (BPL) algorithm which may be advantageous for the signal evaluation of such small lesions, due to better signal-to-noise ratios (SNRs) compared to standard reconstruction techniques.

A potentially relevant difference between ^18^F-FDG-PET and ^68^Ga-PSMA-PET is the positron energy. Positrons emitted by ^68^Ga and ^18^F have a mean energy of 0.88 MeV and 0.25 MeV [16] corresponding to mean ranges in water of 2.9 mm and 0.6 mm, respectively [17]. Higher positron energy negatively affects the spatial resolution, which is well described for high resolution preclinical PET scanners [18,19,20]. For small nodal lesions, the resulting blurring effect may have an effect on measured uptake values and lesion detectability. In addition, PSMA exhibits high specificity causing a high tumor-to-background (T/B) ratio which increases accuracy of quantification for larger lesions and visual detection of small lesions [21,22,23].

The BPL algorithm implemented by GE Healthcare (GE Healthcare, Chicago, IL, United States), Q.Clear, is an iterative reconstruction algorithm which enables users to define a noise penalty factor β. In contrast to ordered subset expectation maximization (OSEM [24]) reconstructions, penalized likelihood reconstructions can be run until full convergence leading to higher quantitative accuracy [25], improved lesion visual conspicuity and maximum standardized uptake value (SUV_max_) in small nodules for low β [26] and a more consistent signal-to-noise ratio [27,28].

Although preferred image smoothness for visual assessment of PET studies is user dependent, suggestions for optimal β values are described in the literature for various types of PET/CT studies: a β of 400 for ^18^F-FDG whole body PET/CT scans [29]; a β of 300 for BPL reconstructions of ^18^F-fluciclovine scans for imaging of recurrent prostate cancer [30] and a β of 4000 for scans after administration of ^90^Y for selective internal radiotherapy [31].

The aim of this study was to explore the effect of acquisition time and reconstruction parameters by providing recovery coefficients for various T/B ratios and sphere sizes, obtained from phantom studies with ^68^Ga-PSMA while applying different β values, and to find an optimal β value for quantification as well as visual assessment of ^68^Ga-PSMA PET/CT scans.

## 2. Materials and Methods

**Figure 1:**
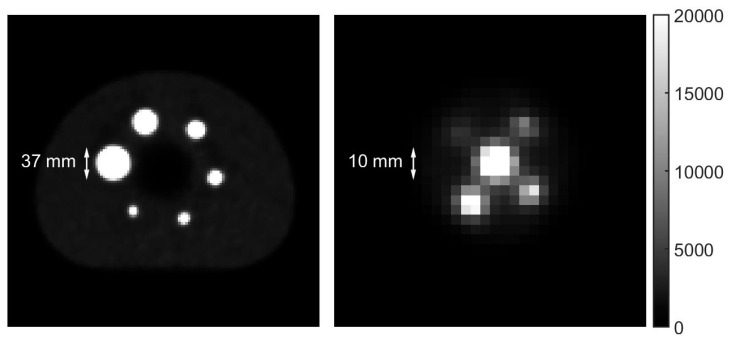
Transverse PET slices of the NEMA IEC Image Quality phantom (**left**) and the Micro Hollow Sphere phantom (**right**). The image of the Micro Hollow Sphere phantom is scaled up by a factor four with respect to that of the NEMA IEC Image Quality phantom to properly show the features. The dimensions of the largest sphere in the Micro Hollow Sphere phantom match with those of the smallest sphere in the NEMA IEC Image Quality phantom. As the voxel dimensions in the transverse plane are 2.73 by 2.73 mm, individual pixels can be clearly distinguished causing a seemingly low image resolution.

The Micro Hollow Sphere phantom (Data Spectrum Corporation, Durham, NC, United States) and the NEMA IEC Image Quality phantom (PTW, Freiburg, Germany) were used to obtain PET/CT images that could be assessed objectively and reproducibly. Both phantoms consist of a fillable background compartment and multiple hollow and fillable spheres with inner diameters 37, 28, 22, 17, 13 and 10 mm for the NEMA Image Quality phantom and 10, 8, 6, 5 and 4 mm for the Micro Hollow Sphere phantom, see Figure 1.

Both the background compartments and sets of spheres were filled with solutions of ^68^Ga in water. To represent a patient scan, the ratio between the activity concentration of both solutions was based on reported T/B ratios for ^68^Ga-PSMA diagnostic PET/CT scans one hour after administration of the radiopharmaceutical. A concise overview is given in Table 1. Based on these reports, the decision was made to perform two scans of the phantoms, one with a ratio of 20:1 and one with a ratio of 10:1 between the activity concentration in the spheres and the background compartment.

### 2.1. Phantom Preparation and Scanning Procedure

Both phantoms were filled in a way similar to the one described in the ‘Standard operating procedures for quality control’ described in the EARL Accreditation Manual [35]. A solution with an activity concentration of 40 kBq/mL used to fill the spheres was prepared by adding 20 MBq ^68^Ga to 500 mL of water (stock solution) and homogenized by extensive shaking. To obtain an activity concentration of 2 kBq/mL in the water-filled background compartments of known volumes, required amounts of ^68^Ga were directly added to these volumes. The solutions in the background compartments were homogenized by shaking the phantoms extensively.

Subsequently, data were acquired with a GE Discovery 710 PET/CT scanner (GE Healthcare, Chicago, IL, USA). Both phantoms were scanned simultaneously. The long axes of both phantoms were aligned to coincide with the axis of the bore. The system was set to acquire data in list-mode to enable multiple reconstructions with different count statistics for both acquisitions. An acquisition time of 10 min per bed position was chosen, with a total of three bed positions per scan. The axial field of view was 15.7 cm and the overlap between subsequent bed positions was 23%. The bed positions were chosen in such a way that the spheres were not placed in the overlapping part of two bed positions.

Directly after the first scan, the activity concentrations in both background compartments were doubled by adding amounts of activity equal to those in step 1, to obtain a 10:1 ratio between the activity concentration in the spheres and the background compartments, correcting for radioactive decay. Again, the background compartments were homogenized by shaking the phantoms extensively. Exactly 68 min (one half-life of ^68^Ga) after starting the acquisition of the first scan, a second acquisition with identical phantom placement and scanning parameters as described in step 2 was performed.

Using the acquired list-mode dataset, multiple iterative reconstructions were made for both scans. All data were corrected for attenuation, random events and scatter. Reconstructions were made with Q.Clear with varying β (300, 400, 450, 500, 600, 700, 800, 900 and 1000) including PSF modelling, for multiple simulated scan times (1, 2, 2.5, 5 and 10 min per bed position). As a reference, conventional iterative OSEM reconstructions with 2 iterations and 24 subsets, 6.4 mm Gaussian filter and 1:4:1 filter in axial direction with and without PSF modelling were obtained. All reconstructions used time-of-flight data and consisted of 2.73 × 2.73 × 3.27 mm^3^ voxels and a 256 × 256-pixel matrix.

### 2.2. BPL Reconstructions

The Q.Clear algorithm introduces a noise control termβR(x) to the objective function used in OSEM reconstructions, where β is the parameter controlling the strength and R(x) is defined as (1):

(1) $$R\left(x\right)=\overset{n_{v}}{\underset{j=1}{\sum }}\underset{k\in N_{j}}{\sum }w_{j}w_{k}\frac{{\left(x_{j}-x_{k}\right)}^{2}}{\left(x_{j}+x_{k}\right)+\gamma \left|x_{j}-x_{k}\right|}$$

where n_v_ refers to the number of voxels, N_j_ is the set of neighboring voxels of voxel j, w_j_w_k_ is the weight of the local smoothing value which depends on the distance between voxels j and k, x is the activity in a voxel and γ is the parameter controlling edge preservation [36].

### 2.3. Background Variability

Background variability (BV) was determined for all reconstructions obtained, based on count statistics in a manually drawn region of interest (ROI) in the background, extended over multiple slices. Care was taken to neither include voxels near the edge of the phantom nor near the hot spheres in order to avoid a bias in the background volume of interest (VOI) due to partial volume effects.

The BV was calculated by (2):

(2) $$\mathrm{BV}=\frac{\sigma_{\mathrm{VOI}}}{\mu_{\mathrm{VOI}}}$$

where σ_VOI_ is the standard deviation of the number of counts in the VOI and µ_VOI_ is the mean number of counts in the VOI.

### 2.4. Activity Recovery Coefficients

The recovery coefficient was used as measure for the ratio between the apparent activity concentration and the true activity concentration in a VOI. Ideally, the RC is equal to 1 for all sphere diameters. In general, the recovery coefficient will gradually decrease for smaller sphere diameters.

RCs were obtained semi-automatically. First, the spheres were identified visually in the PET image. Subsequently a box was manually defined around the maximum voxel value for each sphere. Each box was constructed to fully include a sphere without inclusion of voxels of other spheres. In addition, a background VOI was manually defined in such a way that the boundaries were neither close to the phantom wall nor to the spheres, to ensure homogeneity and avoid partial volume effects.

Next, the maximum voxel value in each box corresponding to a sphere was obtained. The measured ratio R_meas,max_ between the maximum activity concentration C_sphere,max_ in a sphere and the average activity concentration in the manually drawn background VOI C_bg,avg_ (equivalent to the T/B ratio in a patient scan, comparing maximum SUV to the background SUV), was defined as (3)

(3) $$R_{\mathrm{meas},\;\max}=\frac{C_{\mathrm{sphere},\max}}{C_{\mathrm{bg},\mathrm{avg}}}$$

Using the location of the maximum voxel value of each sphere in the PET reconstruction, VOIs to determine the average voxel value in the sphere volume C_sphere,avg_ were constructed automatically using a simple region growing algorithm including all voxels within a 3D isocontour at 50% of the maximum voxel intensity corrected for background [31]. These VOIs were used to calculate the measured ratio between the average activity concentration in the sphere and the background R_meas,avg_ (equivalent to the T/B ratio in a patient scan, comparing mean SUV to the background SUV) (4).

(4) $${\;R}_{\mathrm{meas},\;\mathrm{avg}}=\frac{C_{\mathrm{sphere},\mathrm{avg}}}{C_{\mathrm{bg},\mathrm{avg}}}$$

The peak recovery coefficient RC_peak_ was also determined for each sphere by positioning a spherical contour with a 1.2 cm diameter such that the average voxel value within that sphere is maximized [3]. The measured ratio R_meas,peak_ between the average activity concentration in the spherical VOI C_sphere,peak_ and the background is equivalent to the SUV_peak_ in a patient scan (5):

(5) $$R_{\mathrm{meas},\mathrm{peak}}=\frac{C_{\mathrm{sphere},\mathrm{peak}}}{C_{\mathrm{bg},\mathrm{avg}}}$$

As the actual ratio R between the activity concentration in the spheres and the activity concentration in the background compartments of the phantoms was known, RC_max_, RC_avg_ and RC_peak_ could be calculated by (6)–(8):

(6) $${\mathrm{RC}}_{\max}=\frac{R_{\mathrm{meas},\;\max}}{R}$$

(7) $${\mathrm{RC}}_{\mathrm{avg}}=\frac{R_{\mathrm{meas},\;\mathrm{avg}}}{R}$$

(8) $${\mathrm{RC}}_{\mathrm{peak}}=\frac{R_{\mathrm{meas},\mathrm{peak}}}{R}$$

These RCs are therefore equivalent to the ratios between the observed maximum, average and peak T/B ratio and the true T/B ratio.

Statistical analysis was performed using a Student *t*-test for comparison of data in a single reconstruction and a paired *t*-test for assessment of differences between two reconstructions. A confidence level of 95% was used.

For each sphere, the RC_avg_ values calculated in multiple acquisitions (1 min, 2 min and 5 min per bed position, each with a T/B ratio of 10:1 and 20:1) were averaged and the coefficient of variation (COV) was assessed. The optimal β value was chosen based on reproducibility, i.e., low COV, and detectability, i.e., high recovery and low background variability.

## 3. Results

During the first acquisition, the actual ratios between the activity concentration in the spheres and the background compartments were 20.4:1 and 22.1:1 for the NEMA Image Quality phantom and the Micro Hollow Sphere phantom, respectively. After adding ^68^Ga to the background compartments following the first scan, the second acquisition was performed with phantoms containing activity concentration ratios of 10.1:1 and 11.8:1, respectively.

### 3.1. Background Variability

Background variability was assessed for all available Q.Clear reconstructions. Regarding acquisition parameters, reconstructions from scans with longer acquisition times show lower BV overall due to the higher number of counts and background variability is similar for both scans with different T/B ratios as the background activity concentration is the same. Increasing β results in reconstructions with a lower BV due to the noise reducing effect. In a clinical setting, considering a limited acquisition time, a higher β to obtain less noisy images would be preferable.

### 3.2. Contrast Recovery

#### 3.2.1. NEMA IEC Image Quality Phantom

**Figure 2:**
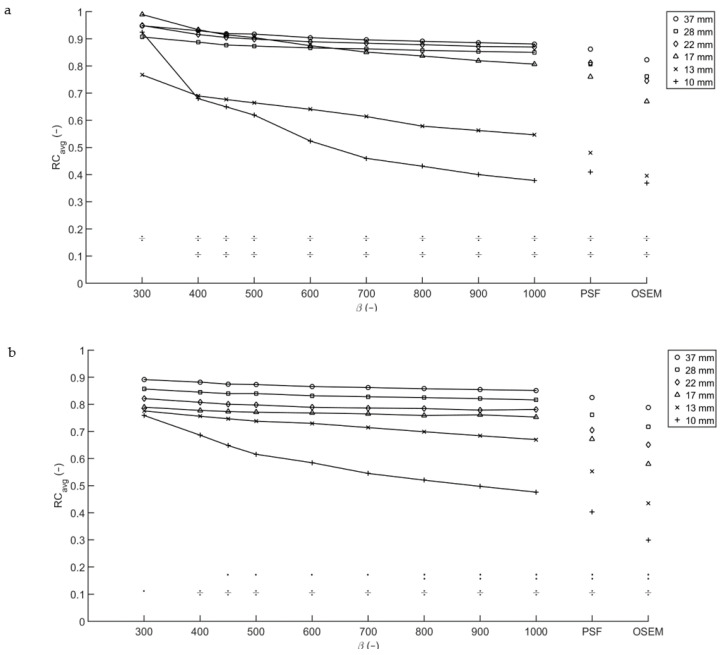
Recovery coefficients from the Image Quality phantom for multiple T/B ratios. RC_avg_ for T/B ratio 10:1 (**a**) and 20:1 (**b**), with acquisition time per bed position of 2 min. The symbols in the lower part of both graphs denote the significance of the differences between the 13 mm sphere and the four biggest spheres (upper row) and the 10 mm sphere and the four biggest spheres (lower row). An obelus (÷) corresponds to *p* < 0.001, a colon to *p* < 0.01 and a single dot to *p* < 0.05.

For a T/B ratio of 10:1, a scan time of 10 min per bed position and a high level of noise tolerance (low β), a relatively constant RC_avg_ between 0.8 and 0.9 is found for the biggest four spheres. The RC_avg_ decreases significantly for spheres with a diameter smaller than 17 mm (*p* < 0.001). Increasing the β to 400 and higher and thus effectively smoothing the image, the decrease in RC_avg_ is already seen in the 17 mm-diameter spheres (*p* < 0.05). Shortening the acquisition time to the clinically used two minutes per bed position resulted in apparently higher average recovery coefficients (Figure 2a). The RC_peak_ of each of the three smallest spheres is lower than that of the three biggest spheres (*p* < 0.001) for both scan times. The higher apparent RCs in the shorter scan do not necessarily correlate with improved lesion detectability due to the increased noise levels.

For the acquisition with a T/B ratio of 20:1 and a scan time of 10 min per bed position, a similar trend was noted. For sphere diameters 17 mm and larger, the average recovery coefficient is similar for all Q.Clear reconstructions. The RC_peak_ of each of the three smallest spheres is lower than that of the three biggest spheres (*p* < 0.001). For the 10 and 13 mm-diameter spheres a spread developed, with a decrease in average recovery coefficient for increasing β. Reconstructions with data acquired for two minutes per bed position (Figure 2b) showed a similar pattern, but with a slightly higher RC_avg_ overall and a more pronounced spread in RC_avg_ for the 10 and 13 mm-diameter spheres.

The four largest spheres with a T/B ratio of 10:1 exhibit a significantly higher RC_avg_ and RC_max_ than those with a T/B ratio of 20:1 (*p* < 0.0001), for all reconstructions considered. For the three biggest spheres, RC_peak_ is similar for both T/B ratios.

#### 3.2.2. Micro Hollow Sphere Phantom

The diameter of the largest sphere in the Micro Hollow Sphere phantom matches with that of the smallest sphere in the NEMA Image Quality phantom. Comparing the two, in general a higher average recovery coefficient is found for the sphere in the Micro Hollow Sphere phantom. These differences in recovery coefficient result from differences in the phantom geometry. An approximate correction factor was introduced to scale the RCs of the Micro Hollow Sphere phantom to those of the NEMA Image Quality phantom. The scaling factor was defined as the ratio between the RC of the matching spheres in the NEMA Image Quality phantom and the Micro Hollow Sphere phantom.

Recovery coefficients are provided for all spheres that could be semi-automatically segmented. For the smaller spheres, the apparent activity concentration in a sphere decreased to less than twice the background value due to the PVE. For these spheres, the region growing algorithm with a threshold 3D isocontour at 50% of the maximum voxel value failed to properly calculate an average recovery coefficient. An increase in β caused a decrease in apparent activity concentration in a sphere and therefore an increase in the number of spheres that could not be properly segmented. A lower T/B ratio also resulted in more difficulties in the segmentation process.

**Figure 3:**
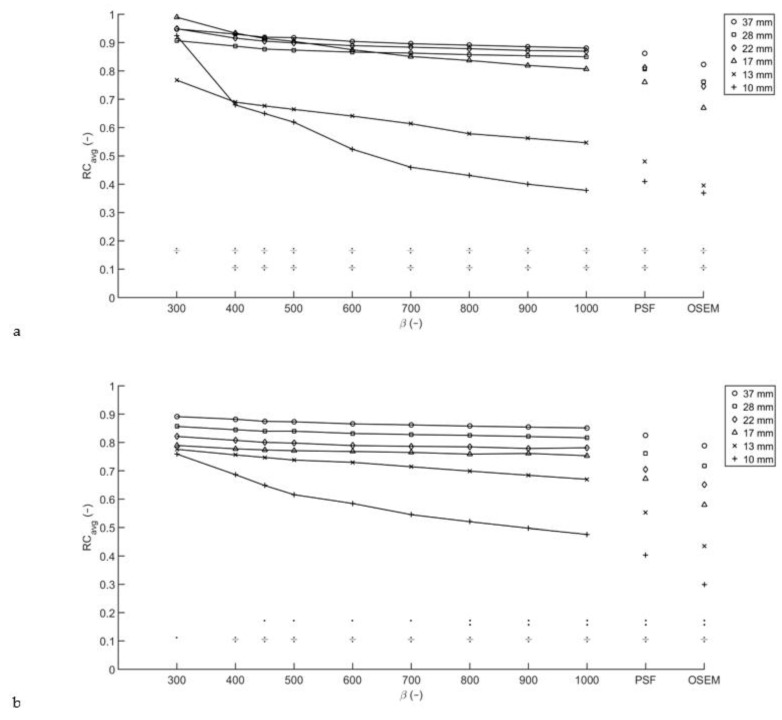
Average and peak recovery coefficients from the Micro Hollow Sphere phantom. For an acquisition time of two minutes per bed position, the apparent RC_avg_ (**a**) of the 8 mm sphere measured with T/B ratio 10:1 exceeds that of the bigger spheres for low β, as the center of this sphere happened to coincide with the center of a voxel. Taking RC_peak_ as a measure for the recovery coefficient (**b**), the recovery coefficients are lower, but more robust.

For both phantom scans performed, a large increase in RC_avg_ for one of the spheres at lower β values was observed as can be seen for the scan with a T/B ratio of 10:1 in Figure 3. Taking RC_peak_ as a quantitative measure, the obtained recovery coefficients appear to be more robust but lower than the RC_avg_.

### 3.3. Reproducibility

**Figure 4:**
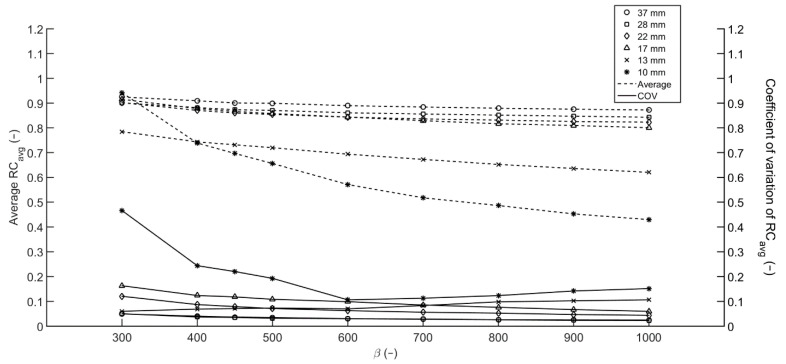
Averaged RC_avg_ and COV from the Image Quality phantom. Averaged RC_avg_ over 6 reconstructions with varying acquisition times per bed position (1, 2 and 5 min) and T/B ratios (10:1 and 20:1).

For each sphere of the Image Quality phantom, the RC_avg_ calculated in the acquisitions with short, medium and long acquisition times (1, 2 and 5 min per bed position), and T/B ratios of 10:1 and 20:1 were averaged and the COV was determined to assess reproducibility considering varying scan parameters. Scans with acquisition times of 2.5 and 10 min per bed position were omitted as these results are similar to 2 and 5 min per bed position, respectively. As shown in Figure 4, the averaged RC_avg_ decreases as β increases, with the largest differences for the RC_avg_ of the smallest sphere. For the largest 4 spheres, the COV decreases as β increases. The COV for the 10 and 13 mm-diameter spheres exhibit an inverse opposite relation as differences in RC_avg_ between the two T/B ratios arise for increasing β. Due to the construction of the prior, the noise penalty term depends on the relative difference in values of adjacent voxels, with higher relative differences yielding better edge preservation. This mainly affects the voxels at the edge of a sphere and hence the RC_max_ and correspondingly the RC_avg_ of bigger spheres is less affected. For spheres consisting of only a few voxels, however, RC_max_ and RC_avg_ will slightly decrease. For the 10 mm-diameter sphere, the minimum COV is found at β = 600. For lower β values, the COV increases as a result of increasing RC_avg_ for shorter acquisition times. This increase corresponds to an increase in RC_max_ which is explained by the higher relative noise level for low count acquisitions. Again, the effect is most profound in small spheres as the number of counts within the region and the maximum number of counts collected in a voxel is smaller than in larger spheres.

## 4. Discussion

Interpretation of SUV metrics is a valuable tool in the assessment of PET/CT scans, as clinically relevant parameters such as d’Amico risk classification, PSA plasma levels and Gleason score correlate significantly with SUV [37,38,39]. However, SUV is also affected by aspects inherent to the imaging method such as uptake time [40], reconstruction algorithm used and the use of PSF modelling [41,42], bed motion [43], use of breathing instructions [44,45], scan time [46] and scanner properties [47]. Therefore, caution is warranted when interpreting SUV for clinical evaluation of ^68^Ga-PSMA PET/CT scans. Differences in pharmacokinetics and pharmacodynamics should be considered when comparing uptake values obtained from scans with different tracers.

Improved lesion conspicuity and increased SUV_max_ for Q.Clear reconstructions with low β are described in the literature [26]. Lowering the β corresponds to less noise suppression and therefore higher SUV_max_ values. For SUV measurements, low β values are found to be more accurate when considering the average uptake in a lesion.

This effect is noticed in phantom scans for measurements of the RC_max_ for both T/B ratios, all simulated acquisition times and all spheres considered in this study. As the RC_avg_ is dependent on the maximum voxel value, this effect is also present in the average recovery curves but less pronounced due to averaging over a larger number of voxels. The RCs exhibited by PSF and OSEM reconstructions are affected by the 6.4 mm Gaussian post-filter, which was chosen based on clinical reconstruction settings in our institute. Lowering or eliminating post-filtering, RCs will increase. On the other hand, even with the post-filter applied, noise levels based on the background variability measurements are higher for PSF and OSEM reconstructions than for any of the BPL reconstructions considered.

The higher recovery coefficients measured for shortened acquisition times are consistent with the increase in SNR. The maximum voxel uptake value is likely to increase when the number of counts is decreased, as the signal-to-noise ratio is proportional to the square root of the number of counts (9):

(9) $$\frac{\mathrm{Signal}}{\mathrm{Noise}}\sim \sqrt{N}$$

Therefore, both the average and the maximum apparent recovery coefficient increase when the number of counts taken into account in the reconstruction is decreased. This effect is less pronounced with increased β, due to the smaller noise tolerance and therefore smoother images from high β reconstructions. In general, caution is needed when comparing SUVs between two scans in which administered activity or scan times differ.

As the two phantoms used in this study were scanned simultaneously, acquisition of the bed position containing the spheres in the Micro Hollow Sphere phantom was started 10 min after acquisition of the bed position containing the spheres of the NEMA Image Quality phantom. Therefore, the activity concentrations in the Micro Hollow Sphere phantom were approximately 6% lower than those in the NEMA Image Quality phantom. The resulting decrease in the number of counts detected probably has a small effect on the maximum voxel value, and may contribute to the difference in recovery coefficients found in the NEMA Image Quality phantom and the Micro Hollow Sphere phantom.

Due to spill-out, RCs are affected by lesion size for smaller lesions. Looking at the sphere diameter at which the spheres’ RC_avg_ deviates significantly from that of the larger spheres in the same reconstruction, a dependence on the β is noted. For higher β, the decrease in RC starts at larger diameters. The volume of each of the three smallest spheres considered in this article (33.51 mm^3^, 65.45 mm^3^ and 113.1 mm^3^) is smaller than five voxels using the minimal voxel size of the used PET/CT scanner (24.37 mm^3^). Coincidental high count rates in a single voxel, for example induced by a coincidental centering of a voxel amid a sphere, can induce a 3D isocontour at 50% of the maximum voxel value that consists of a single voxel. This will result in a positive RC bias, an overestimation of the recovery coefficient.

A large increase in average recovery coefficient observed for the 8 mm-diameter sphere for T/B ratio 10:1 and the 6 mm sphere for T/B ratio 20:1, most evident at low β, is worth mentioning. Detailed inspection of the reconstructions revealed that these spheres appeared to be coincidentally aligned with the reconstruction matrix. As the diameter of the spheres is smaller than three times the minimum voxel dimension, the exact position of the phantom defines the number of voxels over which the total number of counts from the sphere are distributed and therefore strongly influences the recovery coefficient. The effect can be enhanced by a coincidental high number of counts due to Poisson noise, which means the effect is more likely to be noticed for lower β, shorter acquisition times and lower activity concentrations. Taking RC_peak_ rather than RC_avg_ as a measure, the voxel sampling effects are eliminated leading to more robust results. However, as the 1.2 cm-diameter spherical VOI used for obtaining the RC_peak_ is larger than the hot spheres in the Micro Hollow Sphere phantom, this method incorporates background voxels in the VOI, leading to a lower RC. Therefore, in small lesions, SUV_peak_ cannot be used to discriminate between larger volumes with low uptake and smaller lesions with high uptake.

The findings from this study are comparable to those described in ^18^F-FDG PET/CT studies. Improving contrast recovery for lower noise penalties in BPL reconstructions is well described by Teoh et al. [28,29] and similarities between the preferred β values for patient scans in this study and those recently described by Messerli et al. for ^18^F-FDG are also noted [48]; the observation that voxel sampling influences measured uptake values is in line with results for ^18^F-FDG PET/CT shown by Mansor et al. [49] and the observation that RCs decrease for increasing T/B ratio is described by Munk et al. [50]. These similarities are explained by the fact that, from a physics point of view, the main potentially relevant difference between use of ^68^Ga and ^18^F is the positron range.

For a PET system, the spatial resolution can be written as (10):

(10) $$R_{\mathrm{sys}}\approx \sqrt{R_{\det}^{2}+R_{\mathrm{range}}^{2}+R_{180}^{2}}$$

where R_sys_ is the spatial resolution of the system, R_det_ is the contribution of the detectors, R_range_ is the contribution of the root mean square (RMS) positron range in water and *R_180_* is the contribution of the noncollinearity of the annihilation photons [51]. Assuming a system resolution for ^18^F of approximately 5 mm FWHM [15] and evaluating in the RMS positron ranges of 0.23 mm for ^18^F and 1.2 mm for ^68^Ga [52,53], it is evident that the increased positron range only yields an incremental increase in spatial resolution.

To summarize, comparison of SUV measures between different lesions or the same lesion in two different scans is not straightforward even when administration, scanning and reconstruction protocols are equal.

This finding is in line with the conclusion by previous authors that quantitative measures for small lesions in PSF reconstructed PET images can lead to misinterpretation as they vary with lesion size and are less reproducible [50].

Assessment of the reproducibility of RC_avg_ and detectability of lesions in terms of the COV, RC_avg_ and BV for different β suggests a value of 600 as an optimum when quantification as well as detection is of importance. Higher values yield impaired detectability as small lesions blur into the background. Lower values will lead to more accurate uptake measures and better detectability for small lesions. However, the introduction of additional noise will probably yield an increase in false-positives and lower reproducibility which is of particular importance for test–retest studies and follow-up scans.

## 5. Conclusions

Evaluation of PET/CT scans using (semi-)quantitative measures such as SUVs should be performed with great caution, as SUVs are influenced by scanning and reconstruction parameters. Based on the evaluation of multiple reconstructions with different β of phantom scans, an intermediate β (600) is suggested as the optimal value for the reconstruction of clinical ^68^Ga-PSMA PET/CT scans, considering that both detectability and reproducibility are relevant.

## Figures and Tables

**Table 1:** Overview of T/B ratios in several studies concerning ^68^Ga-PSMA PET/CT imaging. The T/B ratio was either computed with the tumor uptake and the SUV_mean_ obtained from a region of interest (ROI) drawn in gluteal muscle [22,32,33] or tumor uptake and the SUV_mean_ from adjacent healthy tissue [34].

	[32] Mean ± SD	[22] Median (Range)	[33] Mean ± SD (Range)	[34] Median (Range)
Lymph node metastases	21.0 ± 27.4		65.2 ± 65.7 (5.3–486.4)	12.2 (3.8–62.2)
Bone metastases	24.7 ± 34.2		84.4 ± 75.1 (3.8–355)	34 (6.8–40)
Local recurrences	15.7 ± 10.1		43.3 ± 33.5 (10.7–144.3)	
Axillary lymph nodes				3 (1.3–8.5)
Primary tumor				18.5 (6.7–92)
Other metastases	16.7 ± 14.1			
Total lesions	21.1 ± 27.4	18.8 (2.4–158.3)		7.8 (1.5–35)

## Data Availability

The data presented in this study are available on request from the corresponding author.

## References

[1] Fitzmaurice C., Allen C., Barber R.M., Barregard L., Bhutta Z.A., Brenner H., Dicker D.J., Chimed-Orchir O., Dandona R., Global Burden of Disease Cancer Collaboration (2017). Global, Regional, and National Cancer Incidence, Mortality, Years of Life Lost, Years Lived With Disability, and Disability-Adjusted Life-years for 32 Cancer Groups, 1990 to 2015. JAMA Oncol..

[2] Mottet N., Bellmunt J., Bolla M., Joniau S., Mason M., Matveev V., Schmid H.-P., Van der Kwast T., Wiegel T., Zattoni F. (2011). EAU Guidelines on Prostate Cancer. Part II: Treatment of Advanced, Relapsing, and Castration-Resistant Prostate Cancer. Eur. Urol..

[3] Boellaard R., Delgado-Bolton R., Oyen W.J.G., Giammarile F., Tatsch K., Eschner W., Verzijlbergen F.J., Barrington S.F., Pike L.C., Weber W.A. (2015). FDG PET/CT: EANM procedure guidelines for tumour imaging: Version 2.0. Eur. J. Nucl. Med. Mol. Imaging.

[4] Fraum T.J., Fowler K.J., Crandall J.P., Laforest R.A., Salter A., An H., Jacobs M.A., Grigsby P.W., Dehdashti F., Wahl R.L. (2019). Measurement Repeatability of 18F-FDG PET/CT Versus 18F-FDG PET/MRI in Solid Tumors of the Pelvis. J. Nucl. Med..

[5] Kurland B.F., Peterson L.M., Shields A.T., Lee J.H., Byrd D.W., Novakova-Jiresova A., Muzi M., Specht J.M., Mankoff D.A., Linden H.M. (2018). Test–Retest Reproducibility of 18F-FDG PET/CT Uptake in Cancer Patients Within a Qualified and Calibrated Local Network. J. Nucl. Med..

[6] Kramer G.M., Frings V., Hoetjes N., Hoekstra O.S., Smit E.F., De Langen A.J., Boellaard R. (2016). Repeatability of Quantitative Whole Body 18F-FDG PET/CT Uptake Measures as Function of Uptake Interval and Lesion Selection in Non-Small Cell Lung Cancer Patients. J. Nucl. Med..

[7] Velasquez L.M., Boellaard R., Kollia G., Hayes W., Hoekstra O.S., Lammertsma A.A., Galbraith S.M. (2009). Repeatability of 18F-FDG PET in a Multicenter Phase I Study of Patients with Advanced Gastrointestinal Malignancies. J. Nucl. Med..

[8] Wang B., Liu C., Wei Y., Meng J., Zhang Y., Gan H., Xu X.-P., Wan F., Pan J., Ma X. (2020). A Prospective Trial of 68Ga-PSMA and 18F-FDG PET/CT in Nonmetastatic Prostate Cancer Patients with an Early PSA Progression During Castration. Clin. Cancer Res..

[9] Zhou X., Li Y., Jiang X., Wang X., Chen S., Shen T., You J., Lu H., Liao H., Li Z. (2021). Intra-Individual Comparison of 18F-PSMA-1007 and 18F-FDG PET/CT in the Evaluation of Patients With Prostate Cancer. Front. Oncol..

[10] Kuyumcu S., Has-Simsek D., Iliaz R., Sanli Y., Buyukkaya F., Akyuz F., Turkmen C. (2019). Evidence of Prostate-Specific Membrane Antigen Expression in Hepatocellular Carcinoma Using 68Ga-PSMA PET/CT. Clin. Nucl. Med..

[11] Moses W.W. (2011). Fundamental limits of spatial resolution in PET. Nucl. Instruments Methods Phys. Res. Sect. A Accel. Spectrometers, Detect. Assoc. Equip..

[12] Soret M., Bacharach S.L., Buvat I. (2007). Partial-Volume Effect in PET Tumor Imaging. J. Nucl. Med..

[13] Kessler R.M., Ellis J.R., Eden M. (1984). Analysis of Emission Tomographic Scan Data: Limitations Imposed by Resolution and Background. J. Comput. Assist. Tomogr..

[14] Hoffman E.J., Huang S.-C., Phelps M.E. (1979). Quantitation in Positron Emission Computed Tomography. J. Comput. Assist. Tomogr..

[15] Van Der Vos C.S., Koopman D., Rijnsdorp S., Arends A.J., Boellaard R., Van Dalen J.A., Lubberink M., Willemsen A.T.M., Visser E.P. (2017). Quantification, improvement, and harmonization of small lesion detection with state-of-the-art PET. Eur. J. Nucl. Med. Mol. Imaging.

[16] Eckerman K., Endo A. (2008). PREFACE. Ann. ICRP.

[17] Bailey D.L., Townsend D.W., Valk P.E., Maisey M.N. (2005). Positron Emission Tomography – Basic Sciences.

[18] Levin C.S., Hoffman E.J. (1999). Calculation of positron range and its effect on the fundamental limit of positron emission tomography system spatial resolution. Phys. Med. Biol..

[19] Cal-Gonzalez J., Vaquero J.J., Herraiz J.L., Pérez-Liva M., Soto-Montenegro M.L., Peña-Zalbidea S., Desco M., Udías J.M. (2018). Improving PET Quantification of Small Animal [68Ga]DOTA-Labeled PET/CT Studies by Using a CT-Based Positron Range Correction. Mol. Imaging Biol..

[20] Disselhorst J.A., Brom M., Laverman P., Slump C.H., Boerman O.C., Oyen W.J.G., Gotthardt M., Visser E.P. (2010). Image-Quality Assessment for Several Positron Emitters Using the NEMA NU 4-2008 Standards in the Siemens Inveon Small-Animal PET Scanner. J. Nucl. Med..

[21] Prasad V., Steffen I.G., Diederichs G., Makowski M.R., Wust P., Brenner W. (2016). Biodistribution of [68Ga]PSMA-HBED-CC in Patients with Prostate Cancer: Characterization of Uptake in Normal Organs and Tumour Lesions. Mol. Imaging Biol..

[22] Afshar-Oromieh A., Malcher A., Eder M., Eisenhut M., Linhart H.G., Hadaschik B.A., Holland-Letz T., Giesel F.L., Kratochwil C., Haufe S. (2013). PET imaging with a [68Ga]gallium-labelled PSMA ligand for the diagnosis of prostate cancer: Biodistribution in humans and first evaluation of tumour lesions. Eur. J. Nucl. Med. Mol. Imaging.

[23] Eder M., Schäfer M., Bauder-Wüst U., Hull W.-E., Wängler C., Mier W., Haberkorn U., Eisenhut M. (2012). 68Ga-Complex Lipophilicity and the Targeting Property of a Urea-Based PSMA Inhibitor for PET Imaging. Bioconjugate Chem..

[24] Hudson H., Larkin R. (1994). Accelerated image reconstruction using ordered subsets of projection data. IEEE Trans. Med Imaging.

[25] Ahn S., Ross S.G., Asma E., Miao J., Jin X., Cheng L., Wollenweber S.D., Manjeshwar R.M. (2015). Quantitative comparison of OSEM and penalized likelihood image reconstruction using relative difference penalties for clinical PET. Phys. Med. Biol..

[26] Howard B.A., Morgan R., Thorpe M.P., Turkington T.G., Oldan J., James O.G., Borges-Neto S. (2017). Comparison of Bayesian penalized likelihood reconstruction versus OS-EM for characterization of small pulmonary nodules in oncologic PET/CT. Ann. Nucl. Med..

[27] Chilcott A.K., Bradley K.M., McGowan D.R. (2018). Effect of a Bayesian Penalized Likelihood PET Reconstruction Compared With Ordered Subset Expectation Maximization on Clinical Image Quality Over a Wide Range of Patient Weights. Am. J. Roentgenol..

[28] Riet J.T., Rijnsdorp S., Roef M.J., Arends A.J. (2019). Evaluation of a Bayesian penalized likelihood reconstruction algorithm for low-count clinical 18F-FDG PET/CT. EJNMMI Phys..

[29] Teoh E.J., McGowan D.R., MacPherson R.E., Bradley K.M., Gleeson F.V. (2015). Phantom and Clinical Evaluation of the Bayesian Penalized Likelihood Reconstruction Algorithm Q.Clear on an LYSO PET/CT System. J. Nucl. Med..

[30] Teoh E.J., McGowan D.R., Schuster D.M., Tsakok M.T., Gleeson F.V., Bradley K.M. (2018). Bayesian penalised likelihood reconstruction (Q.Clear) of 18F-fluciclovine PET for imaging of recurrent prostate cancer: Semi-quantitative and clinical evaluation. Br. J. Radiol..

[31] Rowley L.M., Bradley K.M., Boardman P., Hallam A., McGowan D.R. (2017). Optimization of Image Reconstruction for 90 Y Selective Internal Radiotherapy on a Lutetium Yttrium Orthosilicate PET/CT System Using a Bayesian Penalized Likelihood Reconstruction Algorithm. J. Nucl. Med..

[32] Berliner C., Tienken M., Frenzel T., Kobayashi Y., Helberg A., Kirchner U., Klutmann S., Beyersdorff D., Budäus L., Wester H.-J. (2016). Detection rate of PET/CT in patients with biochemical relapse of prostate cancer using [68Ga]PSMA I&T and comparison with published data of [68Ga]PSMA HBED-CC. Eur. J. Nucl. Med. Mol. Imaging.

[33] Schmuck S., Nordlohne S., Von Klot C.-A., Henkenberens C., Sohns J.M., Christiansen H., Wester H.-J., Ross T.L., Bengel F.M., Derlin T. (2017). Comparison of standard and delayed imaging to improve the detection rate of [68Ga]PSMA I&T PET/CT in patients with biochemical recurrence or prostate-specific antigen persistence after primary therapy for prostate cancer. Eur. J. Nucl. Med. Mol. Imaging.

[34] Sahlmann C.-O., Meller B., Bouter C., Ritter C.O., Ströbel P., Lotz J., Trojan L., Meller J., Hijazi S. (2015). Biphasic 68Ga-PSMA-HBED-CC-PET/CT in patients with recurrent and high-risk prostate carcinoma. Eur. J. Nucl. Med. Mol. Imaging.

[35] EARL Accreditation Manual Version 2.1 (Oct 2020). http://earl.eanm.org/html/img/pool/MASTER_EARL_Manual_Oct2020_2.1.pdf.

[36] Nuyts J., Beque D., Dupont P., Mortelmans L. (2002). A concave prior penalizing relative differences for maximum-a-posteriori reconstruction in emission tomography. IEEE Trans. Nucl. Sci..

[37] D’Amico A.V., Whittington R., Malkowicz S.B., Schultz D., Blank K., Broderick G.A., Tomaszewski J.E., Renshaw A.A., Kaplan I., Beard C.J. (1998). Biochemical Outcome After Radical Prostatectomy, External Beam Radiation Therapy, or Interstitial Radiation Therapy for Clinically Localized Prostate Cancer. JAMA.

[38] Koerber S.A., Utzinger M.T., Kratochwil C., Kesch C., Haefner M.F., Katayama S., Mier W., Iagaru A.H., Herfarth K., Haberkorn U. (2017). 68Ga-PSMA-11 PET/CT in Newly Diagnosed Carcinoma of the Prostate: Correlation of Intraprostatic PSMA Uptake with Several Clinical Parameters. J. Nucl. Med..

[39] Sachpekidis C., Bäumer P., Kopka K., Hadaschik B.A., Hohenfellner M., Kopp-Schneider A., Haberkorn U., Dimitrakopoulou-Strauss A. (2018). 68Ga-PSMA PET/CT in the evaluation of bone metastases in prostate cancer. Eur. J. Nucl. Med. Mol. Imaging.

[40] Beheshti M., Paymani Z., Brilhante J., Geinitz H., Gehring D., Leopoldseder T., Wouters L., Pirich C., Loidl W., Langsteger W. (2018). Optimal time-point for 68Ga-PSMA-11 PET/CT imaging in assessment of prostate cancer: Feasibility of sterile cold-kit tracer preparation?. Eur. J. Nucl. Med. Mol. Imaging.

[41] Lindström E., Sundin A., Trampal C., Lindsjö L., Ilan E., Danfors T., Antoni G., Sörensen J., Lubberink M. (2018). Evaluation of Penalized-Likelihood Estimation Reconstruction on a Digital Time-of-Flight PET/CT Scanner for18F-FDG Whole-Body Examinations. J. Nucl. Med..

[42] Wagner T., Gellee S., Page J., Sanghera B., Payoux P. (2014). Impact of the Point Spread Function on Maximum Standardized Uptake Value Measurements in Patients with Pulmonary Cancer. World J. Nucl. Med..

[43] Yamashita S., Yamamoto H., Nakaichi T., Yoneyama T., Yokoyama K. (2017). Comparison of image quality between step-and-shoot and continuous bed motion techniques in whole-body 18F-fluorodeoxyglucose positron emission tomography with the same acquisition duration. Ann. Nucl. Med..

[44] Bärwolf R., Zirnsak M., Freesmeyer M. (2017). Breath-hold and free-breathing F-18-FDG-PET/CT in malignant melanoma—detection of additional tumoral foci and effects on quantitative parameters. Med..

[45] Li G., Schmidtlein C.R., Burger I.A., Ridge C.A., Solomon S.B., Humm J.L. (2014). Assessing and accounting for the impact of respiratory motion on FDG uptake and viable volume for liver lesions in free-breathing PET using respiration-suspended PET images as reference. Med Phys..

[46] Akamatsu G., Ikari Y., Nishida H., Nishio T., Ohnishi A., Maebatake A., Sasaki M., Senda M. (2015). Influence of Statistical Fluctuation on Reproducibility and Accuracy of SUVmax and SUVpeak: A Phantom Study. J. Nucl. Med. Technol..

[47] Boellaard R., Krak N.C., Hoekstra O.S., A Lammertsma A. (2004). Effects of noise, image resolution, and ROI definition on the accuracy of standard uptake values: A simulation study. J. Nucl. Med..

[48] Messerli M., Stolzmann P., Egger-Sigg M., Trinckauf J., D’Aguanno S., Burger I.A., Von Schulthess G.K., Kaufmann P.A., Huellner M.W. (2018). Impact of a Bayesian penalized likelihood reconstruction algorithm on image quality in novel digital PET/CT: Clinical implications for the assessment of lung tumors. EJNMMI Phys..

[49] Mansor S., Pfaehler E., Heijtel D., Lodge M.A., Boellaard R., Yaqub M. (2017). Impact of PET/CT system, reconstruction protocol, data analysis method, and repositioning on PET/CT precision: An experimental evaluation using an oncology and brain phantom. Med Phys..

[50] Munk O.L., Tolbod L.P., Hansen S.B., Bogsrud T.V. (2017). Point-spread function reconstructed PET images of sub-centimeter lesions are not quantitative. EJNMMI Phys..

[51] Cherry S.R., Sorenson J.A., Phelps M.E. (2012). Physics in Nuclear Medicine.

[52] Derenzo S.E. Precision measurement of annihilation point spread distributions for medically important positron emitters. Proceedings of the 5th International Conference on Positron Annihilation.

[53] Derenzo S.E. (1986). Mathematical Removal of Positron Range Blurring in High Resolution Tomography. IEEE Trans. Nucl. Sci..

