# Correction: Rijnsdorp et al. Impact of the Noise Penalty Factor on Quantification in Bayesian Penalized Likelihood (Q.Clear) Reconstructions of ^68^Ga-PSMA PET/CT Scans. *Diagnostics* 2021, *11*, 847

**DOI:** 10.3390/diagnostics11081371

**Published:** 2021-07-30

**Authors:** Sjoerd Rijnsdorp, Mark J. Roef, Albert J. Arends

**Affiliations:** 1 Department of Medical Physics, Catharina Hospital Eindhoven, Michelangelolaan 2, 5623 EJ Eindhoven, The Netherlands; bertjan.arends@catharinaziekenhuis.nl; 2 Department of Nuclear Medicine, Catharina Hospital Eindhoven, Michelangelolaan 2, 5623 EJ Eindhoven, The Netherlands; mark.roef@catharinaziekenhuis.nl

In the original article [1], there was a mistake in Figure 3 as published. Due to a mistake in the publication process, Figure 2 and Figure 3 were the same. The corrected Figure 3 appears below. The authors apologize for any inconvenience caused and state that the scientific conclusions are unaffected. The original article has been updated.

## Figures and Tables

**Figure 3:**
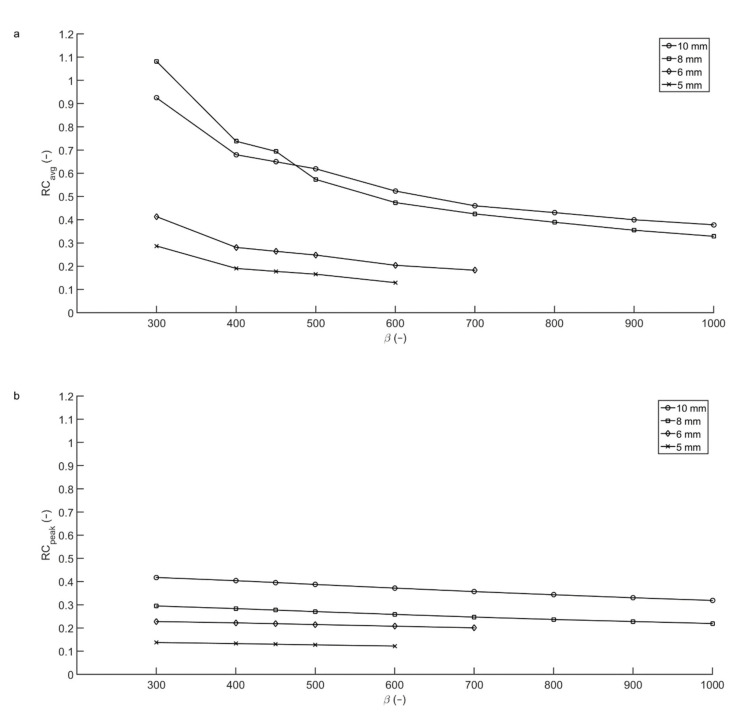
Average and peak recovery coefficients from the Micro Hollow Sphere phantom. For an acquisition time of two minutes per bed position, the apparent RC_avg_ (**a**) of the 8 mm sphere measured with T/B ratio 10:1 exceeds that of the bigger spheres for low β, as the center of this sphere happened to coincide with the center of a voxel. Taking RC_peak_ as a measure for the recovery coefficient (**b**), the recovery coefficients are lower, but more robust.
